# Comparison of Serial and Parallel Connections of Membrane Lungs against Refractory Hypoxemia in a Mock Circuit

**DOI:** 10.3390/membranes13100809

**Published:** 2023-09-24

**Authors:** Albert J. Omlor, Stefan Caspari, Leonie S. Omlor, Anna M. Jungmann, Marcin Krawczyk, Nicole Schmoll, Sebastian Mang, Frederik Seiler, Ralf M. Muellenbach, Robert Bals, Philipp M. Lepper

**Affiliations:** 1 Department of Internal Medicine V—Pneumology, Allergology and Intensive Care Medicine, University Hospital of Saarland, 66424 Homburg, Germany; 2 Department of Anaesthesiology and Critical Care, University Hospital of Saarland, 66424 Homburg, Germany; 3 Department of Internal Medicine II, University Hospital of Saarland, 66424 Homburg, Germany; 4 Laboratory of Metabolic Liver Diseases, Department of General, Transplant and Liver Surgery, Centre for Preclinical Research, Medical University of Warsaw, 02091 Warsaw, Poland; 5 Department of Anaesthesiology and Critical Care, Campus Kassel of the University of Southampton, 34125 Kassel, Germany; 6 Helmholtz Institute for Pharmaceutical Research Saarland (HIPS), Helmholtz Centre for Infection Research (HZI), 66123 Saarbrücken, Germany

**Keywords:** ECMO, mock circuit, serial, parallel, refractory hypoxemia, oxygenator, membrane lung

## Abstract

Extracorporeal membrane oxygenation (ECMO) is an important rescue therapy method for the treatment of severe hypoxic lung injury. In some cases, oxygen saturation and oxygen partial pressure in the arterial blood are low despite ECMO therapy. There are case reports in which patients with such instances of refractory hypoxemia received a second membrane lung, either in series or in parallel, to overcome the hypoxemia. It remains unclear whether the parallel or serial connection is more effective. Therefore, we used an improved version of our full-flow ECMO mock circuit to test this. The measurements were performed under conditions in which the membrane lungs were unable to completely oxygenate the blood. As a result, only the photometric pre- and post-oxygenator saturations, blood flow and hemoglobin concentration were required for the calculation of oxygen transfer rates. The results showed that for a pre-oxygenator saturation of 45% and a total blood flow of 10 L/min, the serial connection of two identical 5 L rated oxygenators is 17% more effective in terms of oxygen transfer than the parallel connection. Although the idea of using a second membrane lung if refractory hypoxia occurs is intriguing from a physiological point of view, due to the invasiveness of the solution, further investigations are needed before this should be used in a wider clinical setting.

## 1. Introduction

Extracorporeal membrane oxygenation (ECMO) is an important rescue therapy method for the treatment of severe hypoxic lung injury which is mainly used in the context of acute respiratory distress syndrome (ARDS) [1,2]. Since the beginning of the COVID-19 pandemic, ECMO therapy has played an important role in the treatment of COVID-19 induced lung injury [1,3,4].

One important challenge is persistent hypoxemia during ECMO therapy, when the system is insufficient to provide adequate oxygenation for a patient [4,5]. In several cases, it can be observed that despite ECMO therapy, the oxygen saturation and the oxygen partial pressure in the arterial blood are low [4,5]. Explanations of the mechanism of refractory hypoxia during ECMO are summarized in Table 1.

One reason for refractory hypoxia can be cannula recirculation, which is defined by the amount of oxygenated blood that is inserted into the right atrium and then directly drained again into the extracorporeal system, bypassing the areas in the body in which oxygenated blood is needed. To reduce recirculation, the distance between the cannulas needs to be maximized or strategies such as the use of a double-lumen cannula or the insertion of two draining cannulas need to be established [5]. Another aspect can be a low hemoglobin value, which requires a blood cell transfusion in order to increase the oxygen-carrying capacity of the blood [5]. However, even with normal hemoglobin and without the recirculation of the cannulas, there can be cases in which a single membrane lung is unable to provide adequate saturation in venovenous extracorporeal membrane oxygenation (VV-ECMO). In these cases, with almost no gas exchange across the lung, the oxygenation mainly depends on the fraction of inspired oxygen (FiO_2_) of the sweep gas, the blood flow of the extracorporeal system and the total gas-exchange surface of the membrane lung. The FiO_2_ is usually already 100% in VV-ECMO and thus cannot be further optimized. The blood flow can also not always be increased as it is limited by the exchange surface of the membrane lung. If the blood flow is too high for a given exchange surface, the contact time in the membrane lung will be too short, and the blood will not be sufficiently saturated with oxygen. According to Bartlett et al., membrane lungs are rated based on the maximum blood-flow rate that can still be completely oxygenated (=post-oxygenator saturation > 95%) [7]. For adults, 7 L rated oxygenators are common, while 5 L rated oxygenators are available for small adults. It has been shown that blood flow needs to be >60% of the cardiac output to provide adequate saturation [8]. Although the use of ECMO during refractory septic shock is controversial and usually involves venoarterial (VA) cannulation [9,10,11], there are case reports of the use of VV-ECMO [4,6]. In sepsis, the cardiac output can rise to 14 L/min and above; in this case, even VV-ECMO with a 7 L oxygenator cannot provide a blood flow rate >60% [6]. In this situation, it would be tempting to combine two membrane lungs to create a more powerful unit.

There is also a second scenario in which the combination of two membrane lungs might be beneficial. Especially at the beginning of the COVID-19-pandemic, supply bottlenecks caused situations in which some membrane oxygenators were available while others were not. In the hypothetic scenario in which 5 L rated oxygenators are available, 7 L rated oxygenators are not available and a single 5 L rated oxygenator is insufficient, one might want to add a second 5 L rated oxygenator in order to provide enough oxygen.

**Figure 1:**
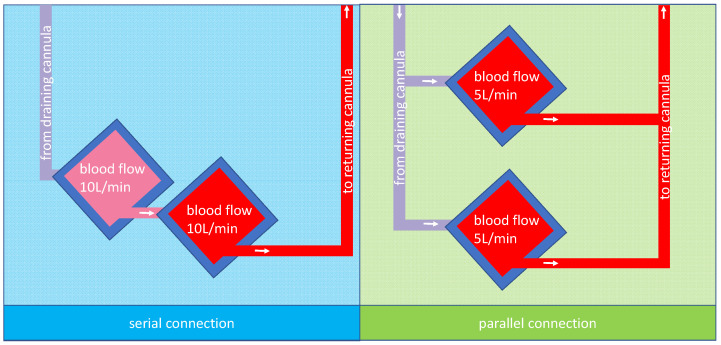
A possible arrangement for membrane lungs: the left panel shows a schematic design of two membrane lungs with integrated centrifugal pumps connected in series and the tubing for the cannulas. Due to the design, the blood flow is the same in both membrane lungs; in this case, 10 L/min. The right panel shows a schematic design of two membrane lungs with integrated centrifugal pumps connected in parallel. In this case, each membrane lung has a blood flow rate of 5 L/min so that in total, a blood flow of 10 L/min is reached.

In both cases, the question would be how to combine two oxygenators. The two possibilities, parallel or serial connection, are shown in Figure 1 for two 5 L rated oxygenators and a total blood flow of 10 L/min.

Patel et al. presented a case series in which two parallel VV-ECMO circuits were introduced in selected patients with elevated cardiac output in COVID-19-related ARDS to increase the blood flow rate. After inserting a second ECMO-circuit in the patient, for each patient, the FiO_2_ on the ventilator could be reduced, the driving pressure for ventilation could be reduced or at least remain the same and the paO_2_ result from an arterial blood gas analysis after 24 h was clearly improved. Out of five cases described in the case series, four patients were successfully weaned from ECMO and discharged from hospital. One of the four patients underwent a double lung transplantation but returned home after 125 days of hospitalization [4].

On the other hand, Kang et al. published a case with a serial connection of two extracorporeal membrane oxygenators for a large patient with refractory hypoxemia. After the connection of the second membrane lung, the hypoxemia improved, the ventilation settings improved, and the patient’s hemodynamics were stabilized. The patient was successfully weaned from both ECMO circuits [12].

Malik et al. presented a case in which two parallel membrane lungs were required in a patient who had a cardiac output between 8 and 10 L/min in sepsis. After the insertion of the second circuit, the arterial partial pressure of oxygen increased clearly, and the FiO_2_ on the ventilator could be reduced from 100% to 30%. The patient was maintained for 14 days on the dual circuit, successfully weaned from ECMO after 60 days and discharged from the hospital later [6].

Despite these case reports, it remains unclear whether the parallel or serial connection is more effective in refractory hypoxic failure during VV-ECMO. Regarding this question, a research of the literature led to a previous study that compared parallel and serial connections of ECMO oxygenators in a porcine animal model [13]. In this study, Melro et al. suggested only a minimal effect on oxygenation of either parallel or serial connection compared to a single ECMO oxygenator with the same blood flow. A difference in the oxygen transfer between a parallel or serial connection was not reported in that study. However, the study design did not reflect refractory hypoxemia in which one ECMO oxygenator is insufficient.

Therefore, we used our ECMO mock circuit, which was designed to measure oxygen transfer rates, to find the best arrangement of two equal membrane lungs in refractory hypoxemia.

## 2. Materials and Methods

### 2.1. Standard Protocol

The ECMO mock circuit described in this work is a simplification of a previously presented mock circuit for full-flow ECMO [14]. In contrast to previous ECMO mock circuits, the new design only requires the onboard sensors of two standard Cardiohelp platforms (Maquet Cardiopulmonar Gmbh, Getinge Group, Rastatt, Germany) to measure oxygen transfer. The mock circuit uses two sequences: one for creating a venous environment with deoxygenated blood, called the regeneration sequence, and the actual measurement sequence.

The mock circuit consists of two 12 L buckets (bucket A and bucket B), a Quadrox-iR adult (Maquet Cardiopulmonar Gmbh, Getinge Group, Rastatt, Germany), a 5 L rated membrane lung with an integrated centrifugal pump at the inlet of the membrane lung and 3/8” polyvinyl chloride tubings, a gas blender with oxygen, carbon dioxide and nitrogen inputs, and two Getinge Cardiohelp platforms (Maquet Cardiopulmonar Gmbh, Getinge Group, Rastatt, Germany) to drive the blood in the circuit and to measure the oxygen saturation, hemoglobin, hematocrit, and blood flow in real time. The measuring range for saturation is from 40% to 100%, and the resolution is 1%. The second Cardiohelp is mainly used for its sensors but can be fitted with a second membrane lung when simultaneous measurements of two membrane lungs in series are required. The blood is tempered to 37 °C with a Maquet HU 35 (Maquet Cardiopulmonar Gmbh, Getinge Group, Rastatt, Germany) via one of the inserted membrane lungs.

As a test fluid, heparinized porcine blood was used. As it is not possible to receive 12 L of blood from one pig, a mix of blood from multiple pigs had to be used. To avoid clotting, 10.000 IE/L of heparin was added, and to avoid bacterial growth, 1 g of meropenem was added. A blood gas analysis was performed at the beginning of the experiment and after 3 h to control and determine the hemoglobin value, acid–base status and lactate and glucose levels. Because no relevant blood deterioration was determined, no adjustments had to be made. The baseline characteristics of the blood in our experiment and the corresponding recommendations for the testing of oxygenators according to the ISO7199:2016 are shown in Table 2.

**Figure 2:**
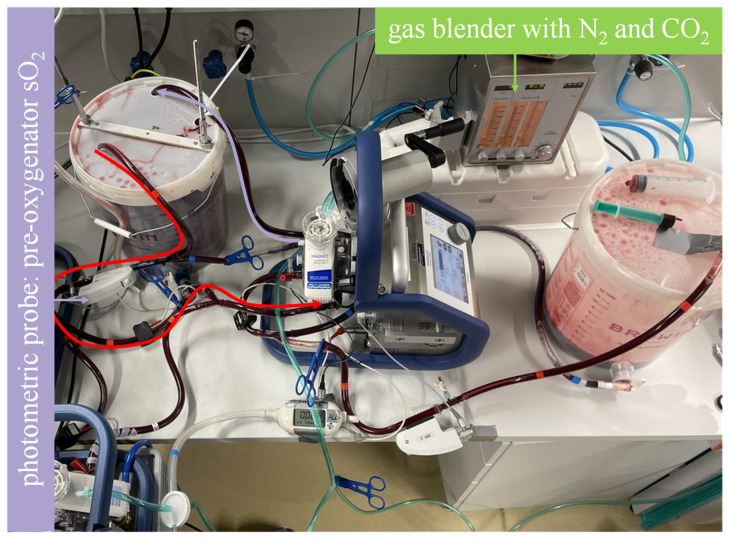
Mock model with blood flow in the regeneration sequence illustrated. As illustrated by the curved arrows, the highly oxygenated blood in bucket A flows through the membrane lung and back to bucket A. A sweep gas comprising N_2_ and CO_2_ achieves deoxygenation and CO_2_ enrichment to establish a venous environment. The photometric probe of one of the Cardiohelp platforms is used to determine when the desired saturation is reached.

In the regeneration sequence (Figure 2), the sweep gas moving through the membrane lung(s) is set to a mixture of N_2_ and CO_2_ at the gas blender. The blood flow is set to move from bucket A through the membrane lung(s) and back into bucket A. A sweep gas flow comprising 8.5 L/min of N_2_ and 0.7 L/min of CO_2_ was chosen to achieve a typical venous environment with a carbon dioxide partial pressure (pCO_2_) of 40 mmHg ± 5 mmHg. Venous saturation is determined in real time via the photometric onboard sensor of the Cardiohelp (Maquet Cardiopulmonar Gmbh, Getinge Group, Rastatt, Germany). The sweep gas flow is stopped once the desired saturation at the pre-oxygenator probe is reached, which in this experiment, was 45%.

After the saturation is reached, the measuring sequence can start. The measurement sequence uses the same membrane lung(s) as the regeneration sequence. The sweep gas is set to 100% oxygen at the gas blender, and the blood flow is redirected from bucket A through the membrane lung(s) to bucket B. For higher accuracy, a mass-flow sensor (TSI41403, TSI GmbH, Aachen, Germany) is connected to the sweep gas outlets of each membrane lung used to verify the sweep flow rate of 5 L/min. The oxygen transfer rate of the membrane lung(s) is calculated from the blood flow, hemoglobin value and the pre- and post-membrane-lung oxygen saturation values. At the end of the measurement sequence, the whole oxygenated and decarboxylated blood is located in bucket B and then transferred back to bucket A. A new regeneration sequence is needed before the next measurement.

The oxygen transfer over a membrane lung is usually calculated as follows:

Q_va_O_2_ = Q_blood_ [dL/min] × (Hb [g/dL] × 1.34 [mL/g] × S_a_O_2_ + p_a_O_2_ [mmHg] × 0.0031

[1/mmHg × mL/dL] − Hb [g/dL] × 1.34 [mL/g] × S_v_O_2_)             

When the physically dissolved oxygen is negligible, such as in this experiment, the formula can be simplified to

Q_va_O_2_ = Q_blood_ [dL/min] × (Hb [g/dL] × 1.34 [mL/g] × S_a_O_2_ − Hb [g/dL] × 1.34 [mL/g] × S_v_O_2_)

### 2.2. Comparison of Serial and Parallel Connections of Membrane Lungs

**Figure 3:**
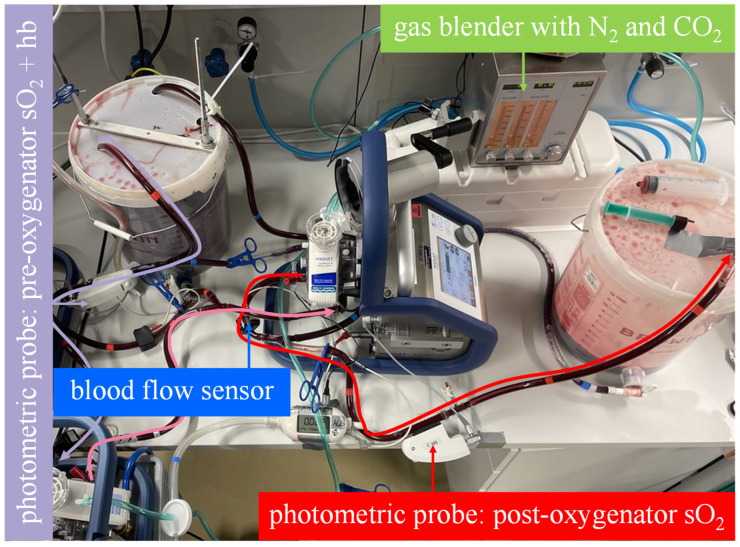
The mock model with an illustrated blood flow in the measuring sequence for the serial arrangement. As illustrated by the curved arrows, the venous blood in bucket A flows through both membrane lungs in series and then into bucket B. The oxygen transfer rate is computed from the blood flow rate, the hemoglobin value and oxygen saturation at the inlet of the first membrane lung and the oxygen saturation at the outlet of the second membrane lung.

**Figure 4:**
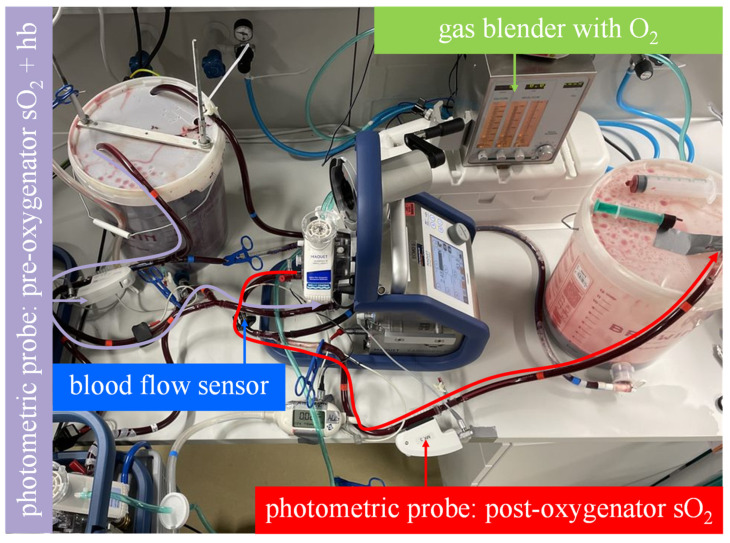
The mock model with an illustrated blood flow in the measuring sequence for the parallel arrangement. As illustrated by the curved arrows, the venous blood in bucket A flows through only one membrane lung at half of the blood flow and then into bucket B. The oxygen transfer rate is computed from the blood flow rate, the hemoglobin value and oxygen saturation at the inlet of the membrane lung and the oxygen saturation at the outlet of the membrane lung. The measured oxygen transfer rate must be multiplied by two to adjust for the second parallel membrane lung that was not measured.

The mock circuit was used to assess the oxygenation rate of two Getinge Quadrox-iR adult 5 L membrane lungs (Maquet Cardiopulmonar Gmbh, Getinge Group, Rastatt, Germany) connected in parallel and serial at a total blood flow of 10 L/min:

1. In the serial arrangement, two equal oxygenators were installed in a row in the mock circuit. Each oxygenator, with its integrated centrifugal pump, was connected to a Cardiohelp platform. The blood flow through the membrane lungs was set at 10 L/min (Figure 3).
2. The parallel arrangement was not physically simulated with two membrane lungs. Instead, the oxygenation rate of one membrane lung was measured at half the total blood flow (5 L/min), and the result was multiplied by a factor of two. This procedure allows the experimenter to quickly switch between parallel and serial arrangements by bypassing just one membrane lung (Figure 4).

### 2.3. Statistics

A statistical analysis was performed using GraphPad Prism 5.02 (GraphPad Software, Inc., La Jolla, CA, USA). Data are presented as medians and interquartile ranges. Differences between groups were analyzed using the Mann–Whitney U test, and *p*-values of <0.05 (*) were considered significant.

## 3. Results

**Figure 5:**
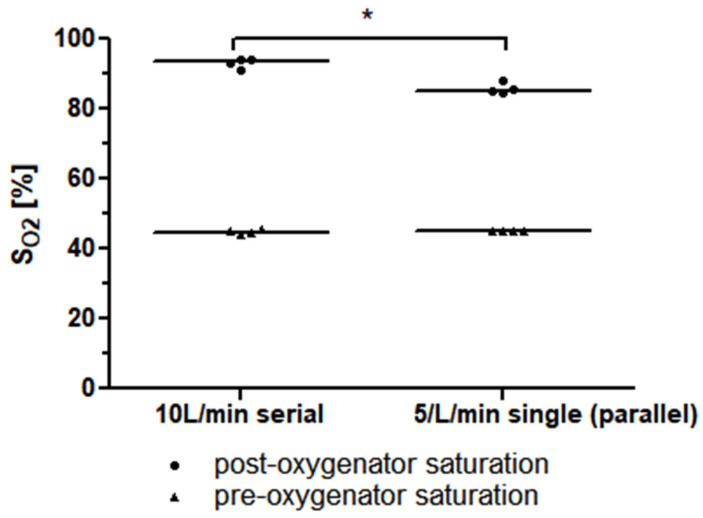
The median pre-and post-oxygenator saturation (S_O2_) values of two identical 5 L rated membrane lungs in a serial connection with a blood flow of 10 L/min is higher than that of a single 5 L rated membrane lung at a blood flow of 5 L/min (or, respectively, two identical 5 L rated membrane lungs in a parallel connection with a total blood flow of 10 L/min).

The required precondition for the simplified mock circuit, a post-oxygenator saturation of lower than 95%, was met for all the measurements. In the measurement of a single Quadrox-iR adult (Maquet Cardiopulmonar Gmbh, Getinge Group, Rastatt, Germany) 5 L rated membrane lung at a blood flow of 5 L/min, a median post-oxygenator saturation of 85.2% (84.9–87.4%) was achieved. The measurement of two Quadrox-iR adult 5 L rated membrane lungs in a serial connection at a blood flow of 10 L/min led to a mean post-oxygenator saturation of 93.5% (91.6–94.2%). The difference in the mean post-oxygenator saturation values was significant (*p* = 0.0286). (Figure 5).

The oxygen transfer rate was 417.4 mL/min (412.1–449.8 mL/min) for a single Quadrox-iR adult 5 L rated membrane lung at a blood flow of 5 L/min. Under the assumption that a second identical membrane lung at the same blood flow of 5 L/min and the same input saturation achieves an equal oxygen transfer rate, a calculated oxygen transfer rate of 834.7 mL/min (824.2–899.7 mL/min) for the parallel arrangement with a total blood flow of 10 L/min is obtained.

**Figure 6:**
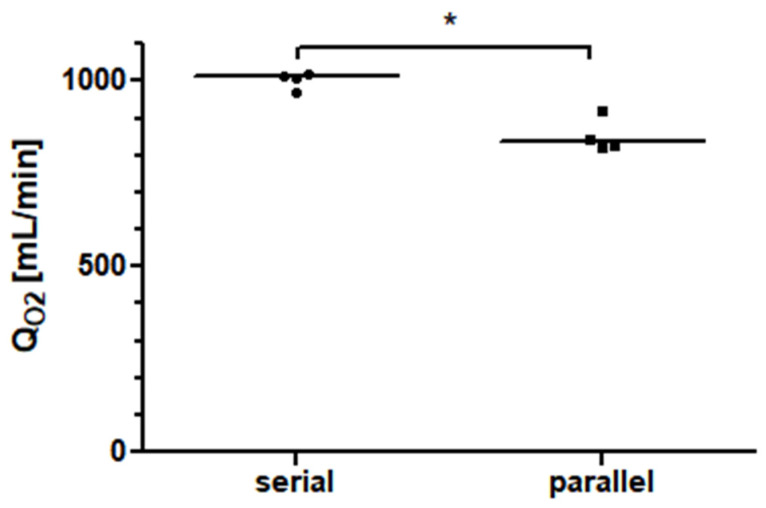
The oxygen transfer rate (Q_O2_) of two identical 5 L rated membrane lungs in a serial connection is about 17% higher than that of the same total blood flow in a parallel arrangement.

Two Quadrox-iR adult 5 L membrane lungs in a serial connection with a blood flow of 10 L/min achieved an oxygen transfer rate of 1010.0 mL/min (981.0–1016.0 mL/min). As shown in Figure 6, at a blood flow of 10 L/min, the measured oxygen transfer rate of the serial arrangement of the two membrane lungs was significantly higher (*p* = 0.0286) than the interpolated oxygen transfer rate of the parallel arrangement. The increase in performance for the serial connection was approximately 17%.

## 4. Discussion

In this work, we present a simplification of an already existing mock circuit for ECMO [14]. The basic idea of building a single-pass model for the measurement sequence and performing the measurements discontinuously, establishing a venous environment first and then performing the measurement, was not changed. However, in contrast to our previous studies, in this experiment, the oxygen transfer rates of the membrane lungs were measured using only the onboard sensors of the Cardiohelp platform rather than the separate CDI Blood Parameter Monitor System 550 (Terumo, Shibuya, Japan) previously used. Because of the simple design and the limitation to mostly common equipment, this simplified ECMO mock circuit could be an entry point into experimental ECMO research for new study groups. We think that at some point in the near future, it might even develop into a viable alternative for animal-based ECMO testing. However, particularly with respect to their ability to model metabolic interactions with organs such as the liver or kidney, mock circuits still have a long way to go. While our model obviously cannot simulate a shock state due to the absence of organs or even vasculature, what we attempted to simulate was an episode of refractory hypoxemia defined by a prolonged state of desaturation despite ECMO. We explain this prolonged state of desaturation with a hyperdynamic cardiac output which overpowers the ECMO flow via dilution, leading to low levels of arterial saturation and a low venous saturation. Those two aspects are both present in our setup.

In clinical ECMO, the blood flow used is typically limited by the cannulas rather than the membrane lungs. Despite this fact, the case reports we found managed to feed two membrane lungs with enough blood flow by placing additional cannulas. Our experiment tried to simulate the scenarios described in those case reports. For simplicity, our model did not include cannulas, but since the case reports mentioned that they achieved such high blood flows of 10 L/min, we assumed that it is possible.

Under normal circumstances, the Cardiohelp platform utilizes a photometric probe which attaches to a window at the blood inlet of the membrane lung inlet to measure the pre-membrane-lung saturation. With some manual skill, a second probe window recycled from a discarded membrane lung can be added to the blood outlet. When the photometric probe of a second Cardiohelp platform is attached there, the post-membrane-lung saturation can be measured as well. The limitation here compared to the CDI Blood Parameter Monitor System 550 (Terumo, Shibuya, Japan) is the lack of the measurement of the post-membrane-lung partial pressure of oxygen (pO_2_) as an indicator of physically dissolved oxygen. That means that the simplified mock circuit can only measure reliable oxygen transfer rates in scenarios in which the physically dissolved oxygen is negligible, which we defined via a post-membrane saturation of less than 95%. Under most clinical conditions, membrane lungs reach a post-oxygenator saturation of 100%. However, we found that a post-oxygenator saturation below 95% can be achieved when the membrane lung is used at its maximum rated blood flow or above and a very low pre-oxygenator saturation of 45% is used. For a post-oxygenator saturation of 95%, the pO_2_ will be somewhere between 75 mmHg and 100 mHg. Even if the higher value is assumed, this translates only into 3 mL/min of additional oxygen transfer, which is easily negligible.

One might argue that the measuring conditions required for our simplified mock circuit are too different to typical clinical scenarios in which the pre-oxygenator saturation is close the central venous saturation and is therefore somewhere near 70%. However, measuring the oxygen transfer rate at the suggested unphysiological conditions has the advantage that the oxygen transfer is almost entirely limited by the oxygenator and not by the uptake capacity of the blood, giving the approach some potential advantages in the benchmarking of membrane lungs.

The second major point of this work is the comparison of the serial and parallel connections of two membrane lungs in cases of refractory hypoxemia on VV-ECMO. We found an advantage of the serial connection. The results from the comparison of the serial and parallel connections in a porcine model from Melro et al. might, at first glance, seem contradictory to our results [13]. However, the authors themselves mentioned that there was a ceiling effect for which the post-oxygenator saturation was 100% in all scenarios. In our opinion, even a single 7 L rated membrane lung used in that experiment was oversized relative to the pig’s oxygen metabolism. In contrast, our experiment simulates a scenario of refractory hypoxemia in which a single membrane lung is insufficient. Unlike Melro et al., we did not compare the serial and the parallel configurations to a single oxygenator. The reason for this was that even with the maximum number of revolutions possible with a Cardiohelp platform (5000 rpm), we were unable to achieve a blood flow of 10 L/min with only a single oxygenator and its single integrated centrifugal pump.

An explanation for the better oxygenation performance of the serial arrangement can be deduced from the concept of the boundary layer [14,15,16,17,18]. The boundary layer is a stationary fluid layer on the outside of the gas fibers that oxygen molecules need to pass via diffusion. An increased blow flow velocity leads to a reduced thickness of this diffusion boundary and therefore a faster oxygen transfer [16]. In the specific experiment, both the serial and the parallel membrane lung arrangements had a total blood flow of 10 L/min. However, only in the parallel arrangement was the total blood flow of 10 L/min divided into a blood flow rate of 5 L/min for each membrane lung. In the serial arrangement, the blood flow remained at 10 L/min in each of the two membrane lungs. Therefore, the blood flow velocity was higher in the serial arrangement and the boundary layer was thinner, which explains the higher oxygen transfer rate.

**Figure 7:**
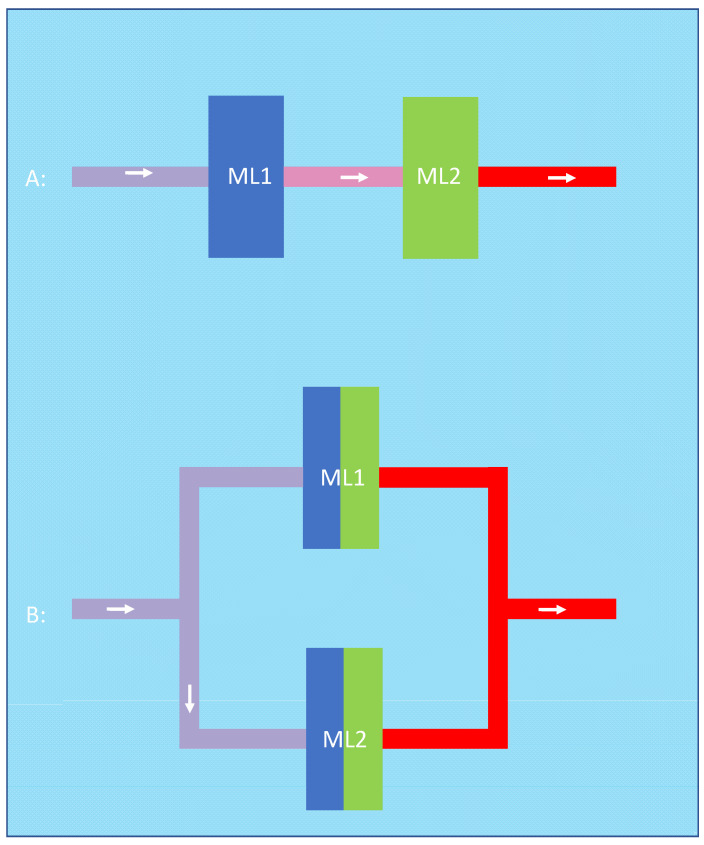
In the serial configuration (A), the first oxygenator (blue) optimizes input conditions such as pH and pCO_2_ for the second oxygenator (green). This can be applied to the parallel configuration (B) in which the two halves closer to the blood inlet (blue) facilitate the oxygen transfer of the two halves closer to the blood outlet (green).

An alternative argument for the advantage of the serial configuration could be that the first membrane lung optimizes the input conditions of the second membrane lung in series. On the other hand, this argument (that the first oxygenator facilitates oxygen transfer by increasing the inlet pH and decreasing the inlet pCO_2_ of the second oxygenator) can also be applied to the parallel configuration. When we imaginarily separate the two parallel membrane lungs into two halves, we can say that the two halves closer to the blood inlet facilitate the oxygen transfer of the two halves closer to the blood outlet. As two halves obviously have the same membrane area as one complete oxygenator, the amount of facilitation should be similar in both cases (Figure 7).

Upon first glance, the currently observed oxygen transfer rate of approximately 1000 mL/min seems unrealistically high compared to the rate of approximately 250 mL/min in our previous full-flow ECMO mock circuit [14]. However, there were multiple factors in the current experiment that led to these high transfer rates. Firstly, the cannula flow was 4× higher (10 L/min vs. 2.5 L/min) than in the previous experiment. Moreover, unlike in the previous experiment, a hemoglobin saturation of 100% was never reached in the current experiment, with its very low pre-oxygenator saturation. Therefore, the oxygen transfer was not potentially limited by an inefficient uptake as physically dissolved oxygen. On top of that, the total effective gas exchange surface area of the two 5 L oxygenators in the current experiment (2 × 1.3 m^2^) was much higher than that of the single 7 L oxygenator in the previous setup (1 × 1.8 m^2^).

## 5. Limitations

Our study has some limitations but also strengths. The data were obtained using a simplified mock circuit. Complex interactions like hemolysis and thrombosis which play important roles during ECMO-therapy could not be investigated. Moreover, the measuring time was limited. On the other hand, the mock circuit allowed us to simulate scenarios of severe refractory hypoxemia that mostly occur in large patients with hyperdynamic circulation. These high ECMO flows often require up to four cannulas. Simulating this in an animal model would not only be technically difficult but would also require very large laboratory animals. Even in our mock circuit, the extremely high blood flow of 10 L/min was challenging because with a reservoir of 12 L of porcine blood, the total measuring time was little more than one minute. Unlike previous experiments, this simplified mock did not allow for the real-time measurement of pH and pCO2, both of which could influence the oxygen-binding curve of hemoglobin. In order to be able to switch quickly between the two configurations in that limited time, the decision was made to not physically simulate the parallel configuration, which would require profound modifications of the mock circuit. Instead, one of the membrane lungs was simply bypassed by changing two clamps, and the blood flow on the remaining membrane lung was reduced from 10 L/min to 5 L/min. We then extrapolated the parallel configuration by multiplying the measured oxygen transfer rate by two, which seems commonsense, as the second parallel membrane lung, which we did not measure, would have had the same input saturation and blood flow as the first one. Moreover, in the clinical case reports that we found, in a parallel configuration, each of the parallel oxygenators typically had their own sets of cannulas, so an interaction between the two oxygenators due to fluid dynamics is very unlikely. Altogether, we therefore concluded that the direct measurement of the parallel configuration was not worth the effort. While the difference between the parallel and serial configurations is statistically significant it is unclear whether the advantage of the serial configuration is clinically relevant. Moreover, a serial configuration with a very high blood flow that requires two centrifugal pumps might also lead to a suction phenomenon at the output of the first oxygenator which might damage and activate the blood. In the worst case, the suction side of the second pump could create such negative pressure on the blood already oxygenated by the first membrane lung that oxygen bubbles form. We think that the risk of bubble formation was rather low in our experimental setting because the first centrifugal pump was set to higher rotations than the second in order to keep the negative pressure low, and the oxygen content of the blood was still low behind the first oxygenator. Nevertheless, we cannot completely exclude that the formation of bubbles might have occurred or even influenced the outcome of our experiment. In a potential clinical application, the risk of the formation of bubbles might be higher as the input saturation could increase as the patient improves. With a higher level of input saturation, the first membrane lung would achieve a higher level of oxygenation, and the risk of bubbles and potentially embolization would increase.

Moreover, as shown in Table 2, our baseline blood characteristics differed in two major aspects from the ISO7199:2016 standard for the testing of oxygenators. As discussed above, the first deviation, the pre-oxygenator saturation of 45% instead of 65 ± 5%, was a requirement for our simplified measuring setup. The second deviation, leaving the hemoglobin concentration at 15.4 g/dL instead of diluting it to 12 g/dL, was employed to keep the setup as simple as possible. Another minor difference from the ISO7199:2016 was the addition of an antibiotic to the blood. Although debatable, we took this precaution in case the blood was contaminated at the slaughterhouse. While these deviations make our data more difficult to compare to other works based on the ISO7199:2016 standard, the internal comparison between the two configurations is valid and might help us gain a better physiological understanding of ECMO.

## 6. Conclusions

The idea of introducing a second membrane lung in an ECMO system when refractory hypoxia occurs is intriguing from a physiological point of view. At least for a very low pre-oxygenator saturation value, a serial arrangement seems to be more effective than a parallel arrangement. However, it was previously demonstrated that both parallel and the serial arrangements typically require very invasive cannulation with two draining and two returning cannulas due to the high levels of blood flow [4,6,12]. Therefore, further investigations are needed before this approach can be used in a broader clinical setting.

## Figures and Tables

**Table 1:** Reasons for refractory hypoxia.

Reasons for Refractory Hypoxia	Potential Mechanisms	Potential Solutions
Cannula recirculation [5]	Highly oxygenated blood from ECMO bypasses the areas in the body in which it is needed	Increasing the distance between the cannulas The use of double-lumen cannulas The insertion of a second draining cannula
Low hemoglobin [5]	A low oxygen-carrying capacity of the blood	Transfusion
High cardiac output [6]	The maximum ECMO flow is <60% of the cardiac output	The use of membrane lungs rated for higher blood flows Combining two membrane lungs

**Table 2:** Blood characteristics at baseline and ISO7199:2016 recommendations.

	Baseline Characteristics	ISO7199:2016 Recommendations
Oxygen Saturation at Membrane Inlet *	45% ± 0.5%	65 ± 5%
Hemoglobin Concentration #	15.4 g/dL	12 ± 1 g/dL
Base Excess #	1.3 mmol/L	0 ± 5 mmol/L
Blood Glucose Concentration #	6.0 mmol/L	10 ± 5 mmol/L
Hematocrit *	48.3%	-
Temperature *	36.1 °C	37 ± 1 °C

*—Data from the Cardiohelp probe during the experiment; #—data from a blood gas sample taken shortly (<5 min) before the experiment.

## Data Availability

Data can be provided upon request to the corresponding author. All data-sharing statements are subject to conformity with German data protection legislation and rules (Datenschutzgrundverordnung—DGSVO).
